# Right bundle branch block: Prevalence, incidence, and cardiovascular morbidity and mortality in the general population

**DOI:** 10.1080/13814788.2019.1639667

**Published:** 2019-07-24

**Authors:** M. Alventosa-Zaidin, L. Guix Font, M. Benitez Camps, C. Roca Saumell, G. Pera, M. Teresa Alzamora Sas, R. Forés Raurell, O. Rebagliato Nadal, A. Dalfó-Baqué, J. Brugada Terradellas

**Affiliations:** a Centre d’Atenció Primària Arenys de Mar, Servei d’atenció Primària Barcelonès Nord- Maresme, Institut Català de la Salut, Barcelona, Spain;; b Centre d’Atenció Primària Berga, Servei d’atenció Primària Bages- Berguedà-Solsonés, Institut Català de la Salut, Barcelona, Spain;; c Centre d’Atenció Primària Gòtic, Servei d’atenció Primària Barcelona Litoral, Institut Català de la Salut, Barcelona, Spain;; d Centre d’Atenció Primària El Clot, Servei d’atenció Primària Barcelona Dreta-Muntanya, Institut Català de la Salut, Barcelona, Spain;; e Faculty of Medicine, University of Barcelona, Barcelona, Spain;; f Unitat de Suport a la Recerca Metropolitana Nord, Institut Universitari d’Investigació en Atenció Primària Jordi Gol (IDIAP Jordi Gol), Barcelona, Spain;; g Centre d’Atenció Primària Riu-Nord Riu-Sud Santa Coloma de Gramenet, Servei d’atenció Primària Barcelonès Nord i Maresme, Institut Català de la Salut, Barcelona, Spain;; h Departament de Cardiologia, Hospital Clínic de Barcelona, Barcelona, Spain

**Keywords:** Right bundle branch block, morbidity, mortality, cardiovascular events

## Abstract

**Background:** Right bundle branch block (RBBB) is among the most common electrocardiographic abnormalities.

**Objectives:** To establish the prevalence and incidence of RBBB in the general population without cardiovascular events (CVE) and whether RBBB increases cardiovascular morbidity and mortality compared with patients with a normal electrocardiogram (ECG).

**Methods:** A historical study of two cohorts including 2981 patients from 29 primary health centres without baseline CVE. Cox (for CVE) and logistic (for cardiovascular factors) regression was used to assess their association with RBBB.

**Results:** Of the patients (58% women; mean age 65.9), 92.2% had a normal ECG, 4.6% incomplete RBBB (iRBBB) and 3.2% complete RBBB (cRBBB). Mean follow-up was five years. Factors associated with appearance of cRBBB were male sex (HR = 3.8; 95%CI: 2.4–6.1) and age (HR = 1.05 per year; 95%CI: 1.03–1.08). In a univariate analysis, cRBBB was associated with an increase in all-cause mortality but only bifascicular block (BFB) was significant after adjusting for confounders. cRBBB tended to increase CVE but the results were not statistically significant. Presence of iRBBB was not associated with adverse outcomes. Patients with iRBBB who progressed to cRBBB showed a higher incidence of heart failure and chronic kidney disease.

**Conclusion:** In this general population cohort with no CV disease, 8% had RBBB, with a higher prevalence among men and elderly patients. Although all-cause mortality and CVE tended to increase in the presence of cRBBB, only BFB showed a statistically significant association with cRBBB. Patients with iRBBB who progressed to cRBBB had a higher incidence of CVE. We detected no effect of iRBBB on morbidity and mortality.

KEY MESSAGES

- In this cohort, almost 8% had RBBB, with a higher prevalence in men and elderly patients.
- The presence of cRBBB seems to tend to increase all-cause mortality and cardiovascular events in comparison with patients with a normal ECG but the adjusted results show no statistically significant differences.
- Patients with iRBBB who progressed to cRBBB had more cardiovascular events.

## Introduction

Right bundle branch block (RBBB) is one of the most frequent alterations of the electrocardiogram (ECG) [1]. When RBBB occurs, one branch delays conducting the electrical impulse and the ventricle is activated by the myocardial propagation of the electrical activity of the other ventricle. Thus, the affected ventricle is depolarized erratically and slowly through an alternative pathway. This delay is shown in the ECG with a widening of the QRS complex (duration >120 ms) and a pattern change, which varies depending on the affected branch.

Many studies have shown that RBBB is a risk factor for cardiovascular (CV) diseases [2] and is associated with CV risk factors (CVRFs) such as hypertension and diabetes mellitus (DM) [3,4]. Some studies have found that RBBB increases CV morbidity and mortality when it coexists with CV diseases. Mortality increases when RBBB appears immediately after acute myocardial infarction (AMI) [5], and the appearance of RBBB in patients hospitalized for exacerbated heart failure (HF) is associated with a worse prognosis [6].

The impact of RBBB in patients with no CV disease has been controversial. Some studies report no risk [3,7,8], whereas others have shown that RBBB increases CV events (CVEs) but the results are not always statistically significant for all the CVEs [9–11]. It has been shown that RBBB is benign in athletes, but more prevalent and associated with a worse outcome after cardiac arrest [12,13]. These studies have limitations: they include only men [7,8,14–16], are hospital-based or the patients had prevalent CVE [5,6,10]. Furthermore, there are no studies involving Mediterranean patients, who have lower cardiovascular morbimortality than Northern European patients [17].

This study aimed to establish the prevalence of RBBB in a general population without cardiovascular history, the incidence of RBBB in a general population without cardiovascular history and a normal ECG at baseline, and whether RBBB, compared to patients with a normal ECG, increases CV morbidity and mortality.

## Methods

### Study design

We performed a historical cohort study with patients from 29 urban primary healthcare centres (PHCs) in the Barcelona city area.

### Selection of study subjects

The study population was composed of two cohorts: The first cohort included patients who had consulted in the Gòtic PHC, Barcelona, Spain and had had an ECG between 2000 and 2015 for any reason. Of 2145 patients, 3614 ECGs were included. Variables were extracted from the computerized clinical records. The ECGs were encrypted to ensure privacy.

The second cohort, a general population cohort, included 3786 patients from the ARTPER multicentre study [18], which analyses the incidence of peripheral arterial disease (PAD) in 28 urban PHCs. The ARTPER study included two ECGs, one at baseline (2006–2008) and one during a follow-up visit (2010–2012). In addition, physical exploration and laboratory test variables were collected. Given the poor state of the baseline ECG records, only 938 were analysed. In the follow-up visit, an ECG was performed on 2532 patients (432 participated in both the baseline and follow-up visits).

ARTPER patients were followed up every six months to review CVE and death. Written informed consent was obtained from all the participants. As ARTPER only included patients older than 49 years, Gòtic patients under this age were excluded. So, the inclusion criteria were patients older than 49 years old; patients had to have undergone at least one ECG (normal or with either incomplete RBBB (iRBBB) or complete RBBB (cRBBB) and have no exclusion criteria.

The exclusion criteria were an unreadable ECG, prevalent cardiovascular morbidity (AMI, angina, cerebrovascular accident (CVA), transient ischaemic attack (TIA), abdominal aortic aneurysm, vascular intervention, HF, PAD, chronic kidney disease (CKD), or an ECG with left bundle branch block, anterior hemiblock, posterior hemiblock, atrioventricular block, signs of ischaemia, Brugada syndrome, atrial fibrillation (AF) or other arrhythmias (atrial, ventricular and supraventricular tachycardia, or escape rhythms).

**Figure 1.**
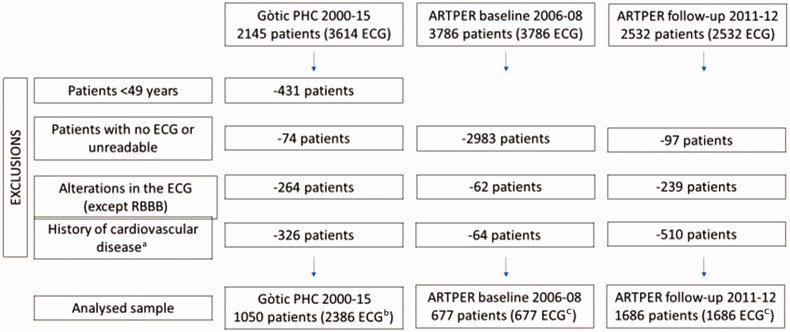
Recruitment of the study population. ^a^Acute myocardial infarction, angina, cerebrovascular accident, transient ischaemic attack, aortic aneurysm, vascular intervention, heart failure, peripheral arterial disease and chronic kidney disease. ^b^1050 patients from Gòtic PHC had 1 to 6 ECG in the period of study. ^c^ARTPER patients performed only one ECG at baseline (245 patients), or only one ECG at the follow-up visit (1254 patients), or at both (432 patients). ARTPER: peripheral arterial disease study; PHC: primary healthcare centre; RBBB: right bundle branch block.

In applying these selection criteria, we finally studied 1050 Gòtic patients (2386 ECGs) and 1931 ARTPER patients (2363 ECGs). In the ARTPER cohort, 1499 patients had only one ECG (245 corresponding to baseline and 1254 to the follow-up visit) and 432 had both the baseline and follow-up visit (Figure 1).

The protocol was approved by the Ethics Committee of the Jordi Gol i Gurina Foundation (registration number FAP 1204).

### Measurements

*ECG reading criteria.* Ten family doctors trained in ECG readings performed the readings. They agreed on the diagnostic criteria for cRBBB and iRBBB, as well as the criteria for other alterations, based on the literature and clinical practice guidelines (Supplementary Table S1).

The definition of RBBB varies greatly among studies. Some use the Minnesota code but others use less strict criteria [3,7,11,14–16,19–21]. We used the more common criteria to avoid missing possible cases (Supplementary Figure S2). To reduce inter-observer variability in reading, the diagnostic criteria were agreed previously with all the researchers. The ECGs were interpreted individually by each researcher, who entered in the database whether RBBB was present or absent, in addition to the other variables mentioned above. The data were later analysed by a statistician who did not know who had interpreted the ECGs.

### Statistical analysis

Categorical variables are described as frequency and percentage, and continuous variables as mean and standard deviation. Prevalence of RBBB was computed using the baseline RBBB cases as numerator and the whole sample as the denominator. Incidence of RBBB was computed using the new RBBB cases at follow-up, only in those with at least two ECG. Person-years were computed as the time between baseline and the first ECG detecting RBBB (cases) or the last ECG (non-cases) for each patient. For iRBBB incidence, patients with a previous RBBB were excluded. For cRBBB incidence, patients with a previous cRBBB were excluded. The association between the baseline presence of RBBB and possible explanatory variables was initially examined by bivariate and multivariate logistic regression, adjusting for potential confounders (age, sex, hypertension, DM, dyslipidaemia, smoking and obesity) and obtaining the odds ratio and 95%CIs. Association between RBBB and CVE (AMI, angina, CVA, TIA, HF, PAD, AF or death) incidence was analysed by Cox regression models adjusted for age, sex and basal comorbidities to obtain the hazard ratios and their confidence intervals. The follow-up time for detecting events began at the time of performing the ECG for patients with presence of RBBB (either at baseline or follow-up) or at the beginning of recruitment for patients who had a normal ECG.

All analyses were performed with 5% significance and two-tailed tests using the Stata statistical package v14 (StataCorp LLC, College Station, TX, USA).

## Results

### Baseline characteristics

Of the 2981 patients in the sample, 58% were women, and the mean age was 65.9 years.

Between the two cohorts, minimal significant differences in age (65.6 vs 66.3 years; *P* = 0.03) and no differences by sex (*P* = 0.375) were observed. All subjects were followed for a mean time of 5.12 years (range: two days to 12.5 years).

Baseline cRBBB was associated independently with male sex and age. The patients with baseline cRBBB also had more hypertension and DM than those with a normal ECG, though the results were not statistically significant. iRBBB was only associated with male sex (Table 1). Obesity, smoking and dyslipidaemia were not associated with either of the two types of RBBB.

**Table 1.** Association of prevalent cRBBB and iRBBB with other cardiovascular risk factors.

Variable	OR	95%CI		*P*
cRBBB				
Men	3.85	2.45	6.06	<0.001
Age (× year)	1.05	1.03	1.08	<0.001
Hypertension	1.52	0.97	2.38	0.067
Diabetes mellitus	1.51	0.95	2.41	0.083
iRBBB				
Men	1.74	1.23	2.47	0.002
Age (× year)	1.00	0.98	1.02	0.866
Hypertension	0.93	0.65	1.33	0.709
Diabetes mellitus	0.90	0.57	1.43	0.660

Logistic regression adjusted for the variables in the table.

### Prevalence of RBBB

A normal ECG was found in 92.2%, iRBBB in 4.5% (*n* = 134/2981; 95%CI: 3.8–5.3) and cRBBB in 3.2% (*n* = 95/2981; 95%CI: 2.6–3.9).

### Incidence of RBBB

iRBBB incidence was 3.5/1000 person-years (py) (*n* = 15 cases/4276 py; 95%CI: 2.0–5.8) and cRBBB incidence was 4.3/1000 py (*n* = 19 cases/4426 py; 95%CI: 2.6–6.7), resulting in an RBBB incidence of 0.8% py.

### Risk analysis of RBBB for CVD outcomes

For AF, PAD, HF, CKD, bifascicular block (BFB) and arrhythmia incident events, we had follow-up information for only 1566 people, with a mean follow-up of 5.12 years, while for death, ischaemic heart disease and CVA we had data for 2750 people, with a mean follow-up of 5.82 years.

After adjusting for age, sex and prevalent comorbidities, only BFB maintained a statistically significant association with cRBBB (HR = 28.66, *P* < 0.001). Patients with cRBBB also had more CVE such as AF, PAD, CVA and HF, but the results were not statistically significant either in the crude models or in the adjusted ones (Table 2).

**Table 2.** Cardiovascular events of patients with RBBB compared with patients with normal ECG (adjusted analysis^a^).

		cRBBB					iRBBB				
		Cases	HR	95%CI		*P*	Cases	HR	95%CI		*P*
Atrial fibrillation	72	6	0.84	0.34	2.09	0.709	4	1.51	0.55	4.17	0.424
Peripheral arterial disease	75	6	1.14	0.48	2.69	0.771	5	1.71	0.69	4.27	0.249
Heart failure	87	7	1.32	0.59	2.95	0.505	3	0.93	0.29	2.96	0.905
Chronic kidney disease	240	10	0.52	0.26	1.02	0.059	13	1.66	0.95	2.91	0.076
Bifascicular block	16	10	28.66	8.48	96.83	**<0.001**	2	1.81	0.24	13.86	0.567
Incident arrhythmia	69	3	0.57	0.14	2.37	0.439	2	0.75	0.18	3.08	0.695
Any of the above	477	30	1.02	0.68	1.52	0.937	21	1.27	0.81	1.98	0.305
Death	224	21	1.47	0.92	2.34	0.110	13	1.31	0.74	2.30	0.352
Ischaemic heart disease	102	4	0.56	0.19	1.60	0.276	6	1.26	0.55	2.89	0.582
Cerebrovascular accident	103	6	0.98	0.42	2.29	0.965	8	1.78	0.86	3.68	0.120

^a^Adjusted by age, gender, hypertension, diabetes and dyslipidaemia.

cRBBB: complete right bundle branch block; iRBBB: incomplete right bundle branch block; HR: hazard ratio.

Bold values refer to statistically significant results.

In patients with iRBBB, no statistically significant association was observed either in the crude models or in the adjusted ones (Supplementary Table S3).

Furthermore, 5.2% (*n* = 7) of patients with iRBBB who progressed to cRBBB showed more cases of HF (HR = 9.54; 95%CI: 1.29–70.57; *P* = 0.007) and CKD (HR = 5.41; 95%CI: 1.33–22.03; *P* = 0.019) than patients who always had a normal ECG, iRBBB or cRBBB.

## Discussion

### Main findings

In the present study of individuals with no known history of CVE, the prevalence of cRBBB was 3.2%. The incidence of RBBB was around 0.8% per year, including both cRBBB and iRBBB. Presence of cRBBB was associated with an increase in BFB.

Although patients with cRBBB had a higher frequency of CVE such as AF, HF, CVA and PAD than those who always had a normal ECG, these results did not reach statistical significance. Conversely, patients with iRBBB who progressed to cRBBB showed more cases of CKD and HF than patients who always had a normal ECG, iRBBB or cRBBB.

### Strengths and limitations

The patients in the study were over the age of 49 years. At younger generations, the presence of RBBB and the incidence of CVE are deficient, so the inclusion of younger subjects did not seem efficient.

Prevalence of RBBB may not represent the general population prevalence. Inclusion of patients referred to an ECG by a GP (only in the Gòtic cohort) may increase this prevalence. Alternatively, exclusion of patients with abnormalities in their ECG or CVD diseases may reduce this prevalence [5–6].

The combination of cohorts to obtain more patients and increase the statistical power meant that the same information was not available on all patients. Nevertheless, some characteristics of the patients, such as hypertension, DM and dyslipidaemia, were similar between the cohorts.

2983/3786 patients from the ARTPER cohort had no ECG readable at ARTPER recruitment. This was due by an administrative cause, leading to a substantial reduction in the ECG available but not causing bias since the loss of these ECGs could be considered at random.

Likewise, the follow-up of events was not homogeneous between the two cohorts in terms of either the available sample (greater in ARTPER for mortality, ischaemic heart disease and CVA) or the validity of the events diagnosed. In the ARTPER cohort, a medical committee confirmed the mortality, ischaemic heart disease, CVA and PAD, while the remaining events and all results of the Gòtic cohort were based only on their unverified appearance in the clinical history. However, when a separate analysis was performed for each cohort, the results were similar to those presented, although the power decreased.

Although the study included 3000 patients, the confidence intervals were relatively wide because of the low incidence of events of interest, the low prevalence of RBBB and the relatively short follow-up.

Despite the limitations, our study is one of the few conducted in a healthy Mediterranean general population.

### Interpretation of the study results in relation to the existing literature

*Prevalence and CVRF.* The prevalence of cRBBB (3.2%) was higher in our study than in other studies [9,11,19]. The higher age of our patients could explain this result. In contrast, the prevalence of iRBBB (4.5%) was lower than in other studies [9]. The prevalence of iRBBB may have been underestimated because the diagnostic criteria used were more stringent than those applied in other studies. Patients with an rsr′ pattern in leads V1 and/or V2 but with a QRS duration <100 ms were not labelled as having iRBBB as defined by the Minnesota code. However, other studies include any QRS <100 ms, so their criterion was broader [3,8,11,16,19,20].

In our study, male sex was significantly associated with greater presence of RBBB, in agreement with previous studies that reported that RBBB is twice as frequent in men as in women [3,7,11,14,16,20].

In the current study, patients with cRBBB had more hypertension and DM, though the results were not statistically significant. Jeong et al. found patients with DM to have a higher risk of RBBB [4]. In the cohort of Thrainsdottir et al. it was observed that in men older than 60 years RBBB is associated with hypertension [3], DM and cardiomegaly, and in women of the same age only with hypertension. The fact that patients with cRBBB have a higher prevalence of CVRF may indicate that the presence of cRBBB is a marker of progression of degenerative cardiovascular disease.

### Incidence of RBBB

The incidence of RBBB was around 0.8% per year. iRBBB incidence was 3.5/1000 py and 4.3/1000 py for cRBBB. The incidence of RBBB was higher in this study than in other studies [4,14]. The higher age of our patients can explain this result.

### Cardiovascular morbidity and mortality

Studies on RBBB and morbidity and mortality show divergent results. The meta-analysis of Xiong et al. studied the impact of RBBB in a general population and in AMI/HF patients [21], concluding that RBBB is associated with all-cause mortality in the general population and in AMI/HF patients, and that RBBB is associated with cardiac mortality in the general population. Bussink et al. observed in their cohort that patients with cRBBB showed greater cardiovascular and all-cause mortality than patients with a normal ECG. Patients with iRBBB showed no increase in morbidity and mortality [11]. Haataja et al. studied the impact of intraventricular conduction delays on cardiovascular mortality with insignificant results related to iRBBB [9]. Likewise, Liao et al. studied healthy men with iRBBB for 20 years and concluded that they had a higher risk of progressing to cRBBB without an increase in secondary morbidity and mortality [8]. Eriksson et al. performed a 28-year follow-up of 70 men with RBBB who showed an increase in high-degree AVB (AV conduction defect II or III) but it was not associated with an increase in secondary mortality [16].

The results of the impact of RBBB on cardiovascular morbidity in other studies vary greatly. Bussink et al. observed that patients with cRBBB have a higher risk of AMI and of need for a pacemaker but not of AF and HF [11]. Schneider et al. studied 70 patients with RBBB from the Framingham cohort and concluded that women with RBBB have a 2.5 and four times greater risk of developing AMI and HF, respectively [14]. This association was not observed in men.

### Implications for clinical practice

The interest and novelty of this study lies in the fact that it was performed in a Mediterranean country whose population has a low cardiovascular risk and in patients treated in primary care, who are the most representative of the general population because most Catalan inhabitants have attended a primary healthcare centre at least once in the last year [22].

Our results show that RBBB does not seem to have an impact on future CVE. Further investigations or referrals to a specialist for asymptomatic patients with RBBB should not be encouraged.

## Conclusion

In this cohort of the general population with no history of CV disease, iRBBB incidence was 3.5/1000 py and 4.3/1000 py for cRBBB, resulting in an RBBB incidence of 0.8% py. Prevalence was higher among men and elderly patients. Although all-cause mortality and CVE tended to increase in the presence of cRBBB, only BFB showed a statistically significant association with cRBBB. Patients with iRBBB who progressed to cRBBB had more CVE. We detected no effect of iRBBB concerning morbidity and mortality.

## Supplementary Material

Supplemental Table 1

Supplemental Figure 2

Supplemental Table 3
