# Supplementary material for: Right bundle branch block: Prevalence, incidence, and cardiovascular morbidity and mortality in the general population
Source: Eur J Gen Pract. 2019 Jul 24;25(3):109–15. doi: 10.1080/13814788.2019.1639667 (PMC6713172; doi:10.1080/13814788.2019.1639667)
Supplement: Supplemental Table 1 [file IGEN_A_1639667_SM8632.docx]

**Table 1: Secondary variables analysed**

| **Variable** | **Description** |
| --- | --- |
| Left bundle branch block | M-shaped QRS complexes (RR') in leads V5, V6, I and aVL.  Wide and slurred S wave in leads V5 and/or V6. QRS complex duration >120ms: complete.  QRS complex duration 100-120 ms: incomplete. |
| Left anterior hemiblock | Marked left axis deviation ≥-30º.  Q1-SII-SIII pattern with wave SIII>SII.  Typical rS pattern in leads II, III, aVF and qR in leads I, aVL. |
| Left posterior hemiblock | Marked right axis deviation >120⁰.  S1-QII-QIII pattern with wave RIII>RII.  Normal QRS complex duration. |
| Bifascicular block | RBBB with LAH: RBBB pattern (QRS >120 ms) + left axis deviation <-30⁰.  RBBB with LPH: RBBB pattern (QRS >120 ms) + right axis deviation >120⁰. |
| Trifascicular block | Bifascicular block plus first-degree AV block. |
| Atrioventricular block | First-degree AVB: constant PR interval with duration >200 ms.  Type I second-degree AVB: progressive lengthening of the PR interval until a beat is dropped.  Type II second degree AVB: intermittent block of AV conduction without lengthening of the PR interval.  Third-degree AVB: complete absence of conduction between the atria and the ventricles. P and QRS complexes follow an independent rhythm. |
| Atrial fibrillation | Absence of P waves.  Presence of irregular F waves (not always visible).  Rapid frequencies and QRS complexes normal and arrhythmic (irregular PR interval). |
| Atrial tachycardia | Frequency >100 bpm. P' waves with different pattern amplitude and axis than sinus waves. |
| Ventricular tachycardia | Presence of >3 consecutive beats with frequency >100 bpm and QRS complex width >120ms. |
| Escape rhythms | Nodal: absence of P waves (may be within or retrograde to the QRS complex). Narrow QRS complexes. Frequency 40-60 bpm.  Ventricular: absence of P waves (may be within or retrograde to the QRS complex). Wide QRS complexes. Frequency 15-40 bpm. |
| Sinus arrhythmia | Variations between two consecutive PP spaces not exceeding 0.18 seconds. Produced by physiological causes (breathing) |
| Sinus bradycardia | Frequency <60 bpm. P wave positive in lead II and negative in lead AVR. Each P wave is followed by a QRS complex (each QRS complex is preceded by a P). |
| Sinus tachycardia | Frequency >100 bpm. P wave positive in lead II and negative in lead AVR. Each P wave is followed by a QRS complex (each QRS complex is preceded by a P). |
| Ectopic heartbeat | Supraventricular extrasystoles: QRS complex with a normal shape that appears before the expected time.  Ventricular extrasystoles: Wide QRS complexes that appear before the expected time and have a compensatory pause. |
| Signs of myocardial necrosis | Presence of a pathological Q wave: Q wave duration >0.04 seconds or Q wave with a voltage >1 mm. |
| Brugada syndrome | Characteristic pattern with pseudoblock of right bundle branch with increase at J-point and decrease of interval ST in leads V1-V2. |
