# Supplementary figures and images for: Right bundle branch block: Prevalence, incidence, and cardiovascular morbidity and mortality in the general population
Source: Eur J Gen Pract. 2019 Jul 24;25(3):109–15. doi: 10.1080/13814788.2019.1639667 (PMC6713172; doi:10.1080/13814788.2019.1639667)

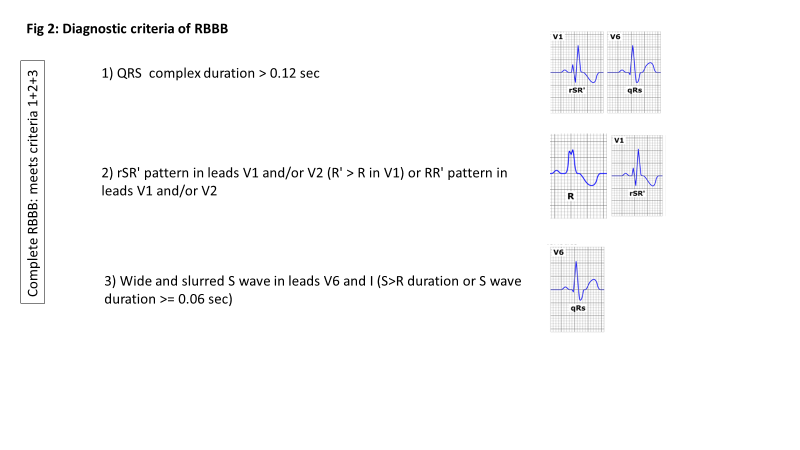

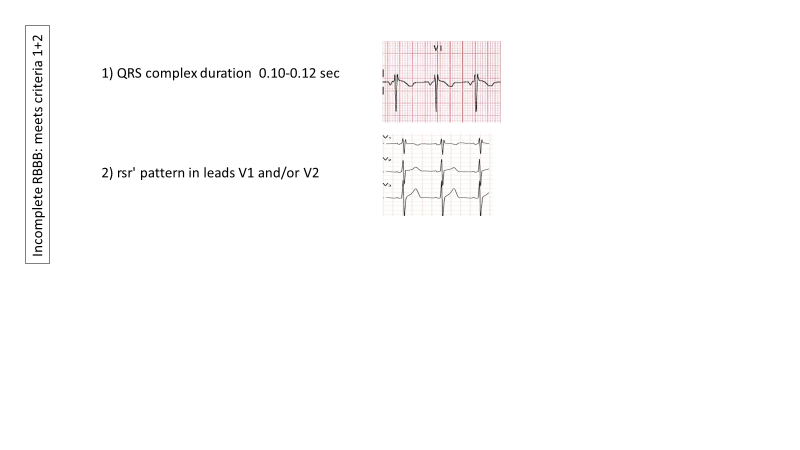

Supplement: Supplemental Figure 2 [file IGEN_A_1639667_SM8611.docx]
