# Supplementary material for: Right bundle branch block: Prevalence, incidence, and cardiovascular morbidity and mortality in the general population
Source: Eur J Gen Pract. 2019 Jul 24;25(3):109–15. doi: 10.1080/13814788.2019.1639667 (PMC6713172; doi:10.1080/13814788.2019.1639667)
Supplement: Supplemental Table 3 [file IGEN_A_1639667_SM8597.docx]

**Table 3: Cardiovascular events of patients with RBBB compared with patients with normal ECG**

|  |  |  |  | **cRBBB** | | | | | | | | **iRBBB** | | | | | | | |
| --- | --- | --- | --- | --- | --- | --- | --- | --- | --- | --- | --- | --- | --- | --- | --- | --- | --- | --- | --- |
|  |  | with RBBB | | Crude analysis | | | | Adjusted analysis* | | | | Crude analysis | | | | Adjusted analysis* | | | |
|  | Cases | **cRBBB** | **iRBBB** | HR | 95% CI | | *P* | HR | 95% CI | | *P* | HR | 95% CI | | *P* | HR | 95% CI | | *P* |
| Atrial fibrillation | 72 | 6 | 4 | 1.46 | 0.63 | 3.41 | 0.379 | 0.84 | 0.34 | 2.09 | 0.709 | 1.58 | 0.57 | 4.34 | 0.376 | 1.51 | 0.55 | 4.17 | 0.424 |
| Peripheral arterial disease | 75 | 6 | 5 | 1.72 | 0.75 | 3.98 | 0.202 | 1.14 | 0.48 | 2.69 | 0.771 | 1.88 | 0.76 | 4.66 | 0.175 | 1.71 | 0.69 | 4.27 | 0.249 |
| Heart failure | 87 | 7 | 3 | 1.63 | 0.75 | 3.53 | 0.217 | 1.32 | 0.59 | 2.95 | 0.505 | 0.96 | 0.30 | 3.06 | 0.951 | 0.93 | 0.29 | 2.96 | 0.905 |
| Chronic kidney disease | 240 | 10 | 13 | 0.68 | 0.35 | 1.32 | 0.253 | 0.52 | 0.26 | 1.02 | 0.059 | 1.63 | 0.93 | 2.85 | 0.088 | 1.66 | 0.95 | 2.91 | 0.076 |
| Bifascicular block | 16 | 10 | 2 | 21.40 | 7.18 | 63.80 | **<0.001** | 28.66 | 8.48 | 96.83 | **<0.001** | 2.06 | 0.27 | 15.72 | 0.485 | 1.81 | 0.24 | 13.86 | 0.567 |
| Incident arrhythmia | 69 | 3 | 2 | 0.56 | 0.14 | 2.28 | 0.415 | 0.57 | 0.14 | 2.37 | 0.439 | 0.75 | 0.18 | 3.08 | 0.694 | 0.75 | 0.18 | 3.08 | 0.695 |
| Any of the above | 477 | 30 | 21 | 1.24 | 0.83 | 1.84 | 0.288 | 1.02 | 0.68 | 1.52 | 0.937 | 1.26 | 0.80 | 1.97 | 0.318 | 1.27 | 0.81 | 1.98 | 0.305 |
| Death | 224 | 21 | 13 | 2.60 | 1.65 | 4.09 | **<0.001** | 1.47 | 0.92 | 2.34 | 0.110 | 1.44 | 0.82 | 2.53 | 0.198 | 1.31 | 0.74 | 2.30 | 0.352 |
| Ischaemic heart disease | 102 | 4 | 6 | 0.99 | 0.36 | 2.70 | 0.984 | 0.56 | 0.19 | 1.60 | 0.276 | 1.34 | 0.58 | 3.05 | 0.493 | 1.26 | 0.55 | 2.89 | 0.582 |
| Cerebrovascular accident | 103 | 6 | 8 | 1.60 | 0.70 | 3.65 | 0.265 | 0.98 | 0.42 | 2.29 | 0.965 | 1.87 | 0.91 | 3.86 | 0.089 | 1.78 | 0.86 | 3.68 | 0.120 |
|  |  |  |  |  |  |  |  |  |  |  |  |  |  |  |  |  |  |  |  |

cRBBB, complete right bundle branch block; iRBBB, incomplete right bundle branch block; HR, hazard ratio.

* Adjusted by age, gender, hypertension, diabetes and dyslipaemia.
